# Fabrication of Hollow Nanocones Membrane with an Extraordinary Surface Area as CO_2_ Sucker

**DOI:** 10.3390/polym14010183

**Published:** 2022-01-03

**Authors:** Waleed A. El-Said, Jin-Ha Choi, Dina Hajjar, Arwa A. Makki, Jeong-Woo Choi

**Affiliations:** 1 Department of Chemistry, College of Science, University of Jeddah, Jeddah 21589, Saudi Arabia; 2 Department of Chemical and Biomolecular Engineering, Sogang University, Seoul 04107, Korea; jhchoi@jbnu.ac.kr; 3 Department of Biochemistry, College of Science, University of Jeddah, Jeddah 21589, Saudi Arabia; Dhajjar@uj.edu.sa (D.H.); Amaki@uj.edu.sa (A.A.M.)

**Keywords:** hollow nanocones membrane, contact angle, CO_2_ sucker, energy storage, polypyrrole, gas sensor

## Abstract

Recently, more and more attention has been paid to the development of eco-friendly solid sorbents that are cost-effective, noncorrosive, have a high gas capacity, and have low renewable energy for CO_2_ capture. Here, we claimed the fabrication of a three-dimensional (3D) film of hollow nanocones with a large surface area (949.5 m^2^/g), a large contact angle of 136.3°, and high surface energy. The synthetic technique is based on an electrochemical polymerization process followed by a novel and simple strategy for pulling off the formed layers as a membrane. Although the polymer-coated substrates were reported previously, the membrane formation has not been reported elsewhere. The detachable capability of the manufactured layer as a membrane braked the previous boundaries and allows the membrane’s uses in a wide range of applications. This 3D hollow nanocones membrane offer advantages over conventional ones in that they combine a π-electron-rich (aromatic ring), hydrophobicity, a large surface area, multiple amino groups, and a large pore volume. These substantial features are vital for CO_2_ capturing and storage. Furthermore, the hydrophobicity characteristic and application of the formed polymer as a CO_2_ sucker were investigated. These results demonstrated the potential of the synthesized 3D hollow polymer to be used for CO_2_ capturing with a gas capacity of about 68 mg/g and regeneration ability without the need for heat up.

## 1. Introduction

Modern civilization needs extensive use of fossil fuels, hence increases greenhouse gas emissions and CO_2_ gas. This is an urgent challenge worldwide because of its effects on global warming [1,2]. CO_2_ gas is emitted from different sources, including power plants, transportation fuels, and other industries such as petrochemicals, iron, steel, and cement [3,4]. The International Panel on Climate Change (IPCC) reported that the average level of CO_2_ would rise to 570 ppm in 2100, which will cause raising the global average temperature by ≈2 °C [2,5]. Among the different techniques for reducing the CO_2_ level, capturing CO_2_ using solid sorbents is a promising technique [6] because it is eco-friendly, cost-effective, noncorrosive, has high gas capacity, and has lower regeneration energy advantages [7,8]. Several solid adsorbents were used for CO_2_ capture, including activated carbons zeolites [9,10,11,12], pillared clays, mesoporous silica [13], and metal–organic frameworks (MOFs) [14,15,16,17]. However, the rapid decline in the adsorption capacities of these adsorbents limited their use. Furthermore, mesoporous silica/amino organic materials [18,19] were applied to capture CO_2_ based on the interaction between primary/secondary amines and the acidic CO_2_ molecules. This interaction results in the formation of a carbamate ion [20,21]. Thus, the development of new materials with high CO_2_ capture efficiency is urgently needed.

Conducting polymers is characterized by a π-conjugated system with promising physicochemical properties. Thus, they have potential applications including biosensors, batteries, corrosion inhibitors, energy storage, solar cells, light-emitting diodes, and electrochemical supercapacitors [22,23,24,25]. Conducting polypyrrole (PPy) showed many advantages, including (i) excellent electric conductivity, (ii) good chemical and thermal stability, (iii) ease of preparation, (iv) good biocompatibility, (v) low-cost effect, and (iv) eco-friendly nature [26,27,28]. Thus, PPy has been widely used as a cell-based sensor [29,30], as a functional material in energy storage [31,32,33], in drug delivery [34,35], as an electroactive material for sensors [36,37], in actuators [38], in artificial muscles [39], in corrosion inhibitors [40], and in solar cells [41,42].

To control the PPy structure’s morphologies, hard porous membranes were used as a hard template for preparing hollow PPy (hPPy) [43,44]. However, several limitations included the high cost, the risk of damage during the hard templates’ removal process, and the complex preparation process. Thus, more effort has been given to the template-free preparation of micro-/nanostructured hPPy film-modified substrates based on the electrochemical technique during the last decade [45,46,47,48,49,50]. The electrochemical polymerization has various advantages, such as a one-step preparation technique, no need to remove the template after the polymerization, and the morphology/properties of the prepared membrane being easy to control [51,52,53]. Although these studies have successfully reported the fabrication of the hPPy nanocone coated layer, it still needed several materials (i.e., surfactants in strongly alkaline media) and complicated synthesizing steps. A simple, in situ, one-step, and controllable template-free electrochemical technique has not been developed to fabricate the hPPy membrane.

Recently, Ag/hPPy/Ag-nanocomposites-modified Au electrodes were reported as surface-enhanced Raman spectroscopy platform for caspase-3 detection [54]. Here, we reported the fabrication of a three-dimensional (3D) hPPy nanocone membrane for the first time, which showed a significantly larger surface area (949.5 m^2^/g) than any previously reported data for hPPy yet. The developed method is simple, in situ, one-step, and controllable template-free electrochemical technique for fabricating the hPPy membrane without a strong alkaline circumstance. The chemical composition and morphology of the prepared membrane were investigated. This membrane was used for CO_2_ capturing that showed a high affiant and hydrophobic characteristic that avoids moisture adsorption.

## 2. Materials and Methods

### 2.1. Materials

Au-coated glass substrates (50 nm of Au/2 nm of Cr/glass wafers) were purchased from G-mek (Korea). Pyrrole and phosphate buffer saline (PBS) (pH 7.4, 10 mM) were purchased from Sigma-Aldrich (St. Louis, MO, USA). Lithium perchlorate (LiClO_4_) was purchased from Janssen Chimica. All other chemicals were obtained commercially as reagent-grade and used without any further purification.

### 2.2. Fabrication of Hollow PPy Nanocone Membrane

Au-coated glass substrates (20 mm × 10 mm, width × length) were cleaned using acidic piranha solution (3:7, H_2_O_2_:H_2_SO_4_) at 70 °C for 5 min. Then, the substrate was rinsed with deionized water (DIW) and ethyl alcohol and dried under N_2_ gas. Electropolymerization of pyrrole to form hPPy membrane was achieved in 0.1 M of the pyrrole containing 0.1 M of LiClO_4_. The cyclic voltammetry (CV) was applied within a potential range from −0.8 V to +1.2 V at a scan rate of 100 mV/s vs. Ag/AgCl electrode [54]. The active area for the electrochemical polymerization of pyrrole to form hPPy nanocones over the Au electrode was 10 mm × 10 mm. However, substrates with larger sizes were also used for some experiments. Furthermore, different concentrations of pyrrole monomer were used to study the effect of the monomer concentrations on the morphology of the resulting polymer. Here, we have shown the effect of three pyrrole concentrations, 0.001, 0.01, and 0.1 M. The DTG-60 Simultaneous Thermogravimetry/Differential Thermal Analyzer (Shimadzu) was used to study the thermal gravimetric analysis (TGA) under air atmosphere. The X-ray diffraction (XRD) of the hPPy polymer was obtained using X-ray PW 1710 control unit Philips anode material Cu (40 K.V, 30 M.A) Optics (Flex Ltd., Friesland, Netherlands): Automatic divergence slit. Furthermore, the Fourier transform infrared spectroscopy (FTIR) spectrum of the prepared polymer was measured using Nicolet 6700 Thermo Fisher Scientific USA spectrophotometer, using the KBr pellet technique. 

### 2.3. Electrochemical Polymerization 

The hPPy was prepared based on electrochemical polymerization using a potentiostat (CHI-660a, CH Instruments, Austin, TX, USA) controlled by Nova software. The electrochemical measurements were performed using a homemade three-electrode system consisting of a bare Au electrode as a working electrode, a platinum wire as the counter electrode, and an Ag/AgCl reference electrode at a scan rate of 100 mV/s. The morphologies of the hPPy films were studied using field emission scanning electron microscopy (FESEM). The FESEM images were recorded using the ISI DS-130C instrument (Akashi Co., Tokyo, Japan). For better capturing the SEM images of samples, the substrates were fixed on the SEM stage with carbon tapes. Pt films were deposited onto the surface of the substrate at room temperature. The sputtering deposition was performed for 15 s under a constant deposition rate. Then, the substrates were being placed into the FESEM chamber. For the cross-section image, a 45-degree stage was used.

### 2.4. CO_2_ Capture Performance

The CO_2_ capture efficiency of the hPPy membrane was studied using thermogravimetric analysis (TGA) in the presence of pure CO_2_ gas at 50 °C. Typically, the platinum sample pan of the TGA was charged with 10 mg of hPPy and kept the temperature at 50 °C for 30 min under pure N_2_ gas to remove any moisture from the hPPy. The CO_2_ adsorption was performed by switching the gas from N_2_ to CO_2_ (99.9%) for a further 60 min. Then, it was switched back to N_2_ to achieve the desorption process at the same temperature for 60 min. The CO_2_ uptake capacity was determined based on the sample’s weight change during the sorption/desorption processes measured using TGA.

## 3. Results and Discussion

### 3.1. Subsection

**Figure 1:**
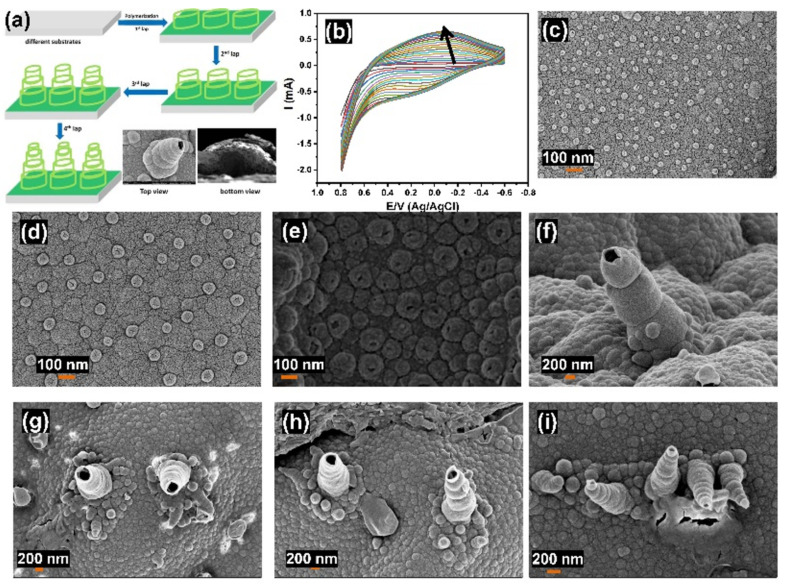
(**a**) Schematic diagram for growing polymer hollow nanocones, (**b**) electrochemical polymerization of PPy, (**c**–**e**) SEM images of polymer nanocones growing based on monomer concentration (0.001 M, 0.01 M and 0.1 M), and (**f**–**i**) effect of the number of cycles on the morphology of polymer nanocones.

Recently, hPPy film-modified substrates were reported in the presence of a soft template in strongly alkaline media based on the electrochemical polymerization process [52,53]. Furthermore, we have reported on the fabrication of hPPy-modified Au electrodes without any linker or template as a surface-enhanced Raman spectroscopy platform for caspase-3 detection [54]. Here, we have prepared hPPy films based on a one-step and easy method using electrochemical polymerization without any surfactants followed by a pull off the film, as shown in Figure 1a. In this schematic diagram, nanocones were growing with the increasing cyclic number of electropolymerization. Figure 1b showed the CV behavior for the pyrrole electropolymerization process for 20 cycles. From the CV data at the beginning of the polymerization process, the CV showed a cathodic peak at about −0.28 V and an anodic peak at +0.0 V. These redox peaks disappeared, and a new cathodic peak at about −0.5 V and anodic peak at about +0.1 V were observed during the polymerization process, combined with increasing background potential. These results mean that progress polymerization of hPPy was correlated to electrical conductivity. Figure 1c–e showed the SEM images of the hPPy film formed after 5 cycles using 0.001 M, 0.01 M, and 0.1 M of pyrrole, respectively. The results demonstrated the fabrication of a layer of mono-laps (nanospheres) with varying sizes diameters based on the monomer concentrations. Increasing the cyclic numbers result in more laps and nanocone structures being formed, as shown in Figure 1f–i. These results confirmed that the nanocones were fabricated based on a lap-over-lap technique that included growing the first stage as hollow nanospheres (first lap). Then, another lap was repeatedly developed over the previous one with each cycle to form nanocones structures.

**Figure 2:**
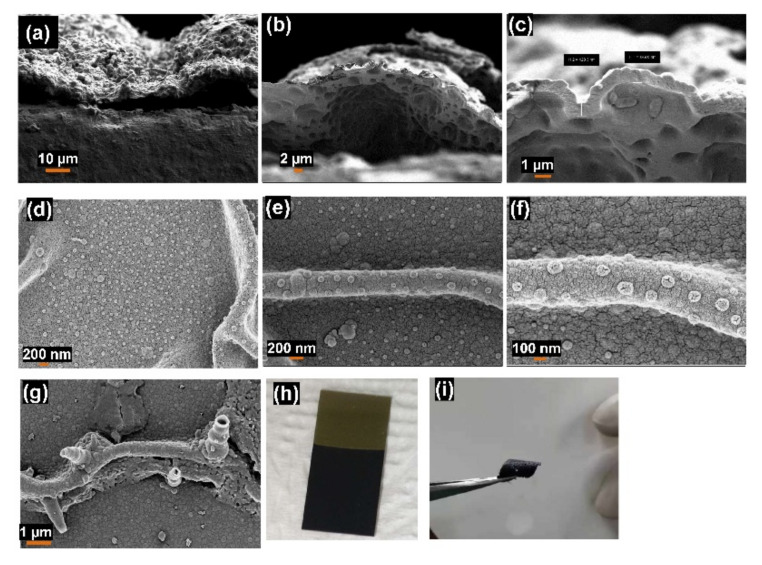
(**a**–**c**) SEM images of a cross-section of the hPPy membrane that showed the morphology of polymer nanocones from the bottom, (**d**–**g**) SEM images of vertical nanocones and horizontal channels (connections), and photography images hPPy nanocones membrane before (**h**) and after (**i**) pull-off from Au substrate.

The morphology of the membrane’s bottom was investigated using SEM images, as shown in Figure 2a–c, which confirmed that the cones are opened from both sides. These results illustrated that this polymer layer did not form as a single domain of hollow nanostructures. Numbers of domains were created in which each one was surrounding with a channel or connection. Furthermore, hollow structures (spheres or cones) were also formed over these channels or connections as shown in Figure 2d–g.

**Figure 3:**
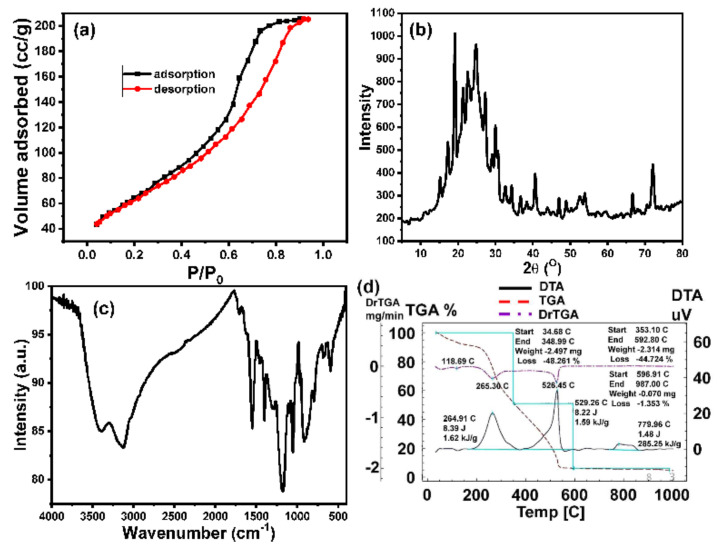
(**a**) N_2_ adsorption/desorption isothermal (surface area) of hPPy nanocones membrane, (**b**) XRD pattern, (**c**) FTIR of hPPy nanocones membrane, and (**d**) TGA of hPPy nanocones membrane.

This contrasts with the previous studies for the synthesis of hollow polymer-modified substrates [52,53,54], which assumed that these polymers have a large surface area. However, it is not measurable because it was obtained only as a film on a solid substrate. Here, the formed hPPy polymer film possesses an exciting advantage that concerns its detachable advantage on an easy pull-off technique, as shown in Figure 2h,i and Video S1. Therefore, the surface area of the resultant polymer membrane was measured based on the N_2_ adsorption/desorption technique (Figure 3a). The Brunauer–Emmett–Teller (BET) method was used to calculate the surface area of the prepared materials. The obtained hollow nanocone polymer membranes show a large surface area of about 949.5 m^2^/g. Table 1 listed the previously reported surface area of hPPy compared to that of the present polymer. This data confirmed that the present polymer has the greatest surface area than the reported surface areas for hPPy [55,56,57,58,59]. The adsorption hysteresis (Figure 3a) exhibits type IV isotherm [60,61,62], which confirmed the presence of mesoporous material. The isotherm exhibits hysteresis loops which are attributed to the presence of mesopores in the obtained materials. This H1 hysteresis loop (IUPAC classification), which implies the presence of porous materials consisting of well-defined cylindrical-like pore channels or agglomerates of approximately uniform spheres [60,61,63].

Furthermore, the bottom of the membrane’s morphology was investigated using the SEM image (Figure 2a–c), which confirmed that the cones are opened on both sides.

The XRD and the FTIR techniques were used to investigate the chemical composition of the developed hPPy membrane (Figure 3b,c), which show the characteristics peaks for the hPPy. The FTIR spectrum showed several bands at the wavenumbers of 1460 and 1550 cm^−1^ (symmetric and asymmetric C–C stretching vibrations), 1300 cm^−1^ (C–N stretching vibration), and 1050 cm^−1^ (bending vibration of the C–H bond). In addition to a broadband in the range from 3000 cm^−1^ to 3500 cm^−1^ (the adsorbed H_2_O and N-H of the pyrrole ring) [64]. X-ray diffraction spectra have shown that pure polypyrrole is amorphous with a broad peak centered at around 2θ = 24.84° [65]. Furthermore, the appearance of peaks at 32.68°, 36.76°, and 38.32° [JCPDS: 30-0751] confirmed the presence of LiClO_4_ inside the hPPy film [66].

The thermal stability of the prepared hPPy membrane was investigated using TGA (Figure 3d). The results demonstrated that the membrane is degraded in two steps. The first stage started from 35 °C to about 249 °C that showed a weight loss of about 10%, which was related to the water loss from hPPy. The second stage ended at 600 °C, which was attributed to the thermal degradation of the hPPY backbone.

**Figure 4:**
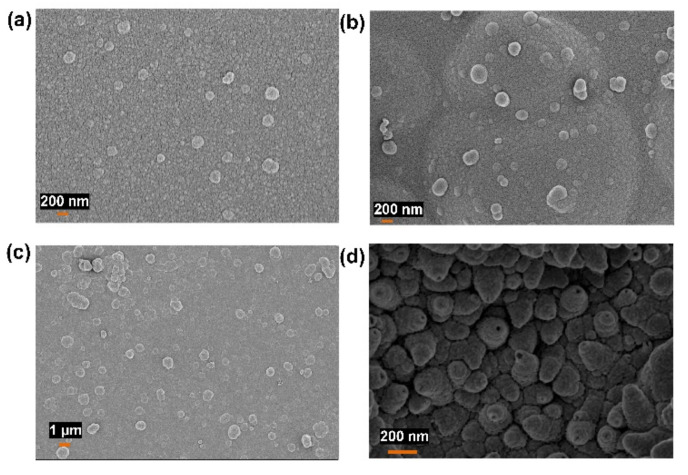
SEM images showed the morphology of hPPy that formed in the presence of (**a**) oxalic acid, (**b**) HClO_4_, (**c**) H_2_SO_4_, and (**d**) LiClO_4_ as counterion ions.

**Figure 5:**
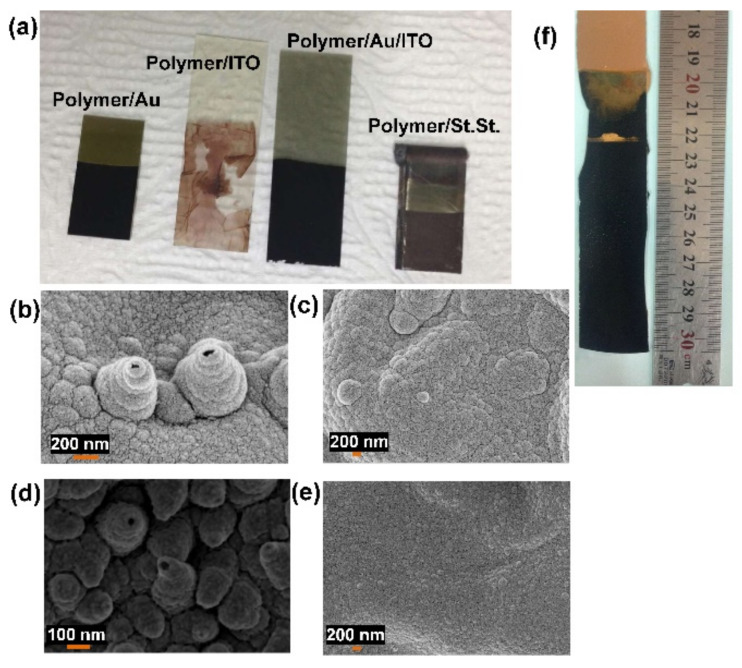
(**a**) Photography images hPPy on different substrates (Au, ITO, Au/ITO, and stainless steel substrates), (**b**–**e**) SEM images of hPPy on Au, ITO, Au/ITO, and stainless steel substrates, respectively, and (**f**) photography image of a large scale of hPPy-nanocones-modified Au substrate.

To investigate the effects of various conditions on the resultant membrane morphology and its different futures, various counterions, supported substrates, and different types of monomers were used. Oxalic acid, HClO_4_, and sulfuric acid were used as counterions instead of LiClO_4_. The SEM images of the hPPy membrane formed in the presence of different counterions are represented in Figure 4, which demonstrated that the hollow structures were obtained only when LiClO_4_ was used as a counterion. The effect of the chemical composition of the used supporting substrate was investigated using different substrates including indium tin oxide (ITO), Au-coated glass, Au/ITO, and stainless steel (SS), as shown in Figure 5a. The SEM images of the hPPy layer on the different substrates were represented in Figure 5b–e, which demonstrates that the hPPy layer has good adhesion with Au, SS, and Au/ITO substrates. In contrast, it has a low adhesion with ITO substrate. The morphology of the resulting polymer also depends on the substrate used. After optimizing all the preparation conditions, the hPPy membrane was prepared on a large scale of Au/glass substrate (3 cm × 10 cm), as shown in Figure 5f. The results revealed the fabrication of hPPy over a 30 cm^2^ area. Hence, we applied the mass production technique with a recyclable advantage for the used substrate numerous times after removing the membrane.

**Figure 6:**
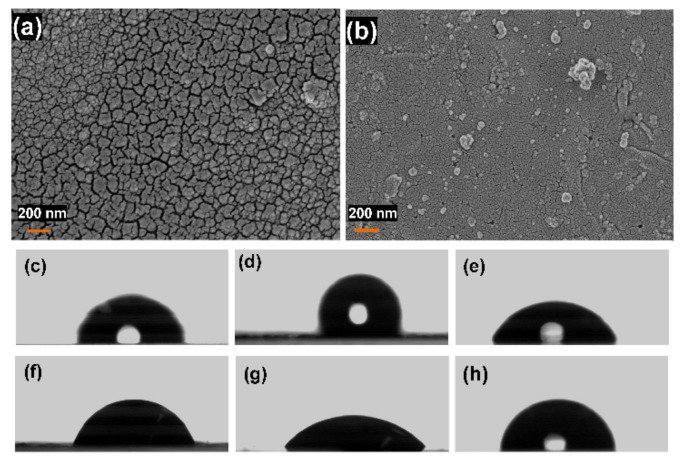
SEM images of (**a**) SEM images of PANI/LiClO_4_, (**b**) SEM images of Au/PANI-modified Au substrate, (**c**) the contact angle of bare Au substrate, (**d**) the contact angle of hPPy-nanocones-modified Au substrate in the presence of LiClO_4_, (**e**) the contact angle of hPPy-nanocones-modified Au substrate in the presence of oxalic acid, (**f**) the contact angle of hPPy-nanocones-modified Au substrate in the existence of HClO_4_, (**g**) the contact angle of hPPy-nanocones-modified Au substrate in the presence of H_2_SO_4_, and (**h**) the contact angle of PANI/Au substrate.

In addition, electropolymerization was performed using the aniline monomer instead of pyrrole. The SEM images of the resultant polymer were represented in Figure 6a,b. These show a thin layer of the polymer with strong adhesion properties but no cone-like structures. Based on these results for constructing the polymer nanocone membrane, pyrrole is the best monomer in the presence of LiClO_4_ as a counterion with a supporting substrate such as Au-coated glass. Interestingly, the formed hPPy membrane possesses a huge surface charge that results in a repulsion force between the species of the hPPy membranes. Furthermore, this surface charge causes an attractive force between the hPPy membrane and the plastic materials, as shown in Videos S2–S4. Hence, the hPPy membrane could be moved using this kind of attraction force, which opens the door for using this hPPy membrane to develop artificial muscles.

The morphology of the prepared hPPy nanocones is like the lotus flowers. Hence, it was expected that hPPy would show a high hydrophobic characteristic. Thus, the contact angle between water and the hPPy-nanocones-modified Au substrates was studied. Figure 6c–h showed the images of the water contact angles with (i) different-hPPy-layers-modified Au substrate formed in the presence of different counterion ions, (ii) the Au/hPPy/Au-modified Au substrate, and (iii) PANI/Au substrate in comparison with the bare Au substrate. These data indicated that hPPy nanocones/Au formed in the presence of LiClO_4_ have the highest contact angle (136.3°). Thus, it possesses a high hydrophobic characteristic. This hydrophobic characteristic was decreased after Au deposition (60.6°), while the bare Au showed a contact angle of about 73.1°.

### 3.2. CO_2_ Capturing

**Figure 7:**
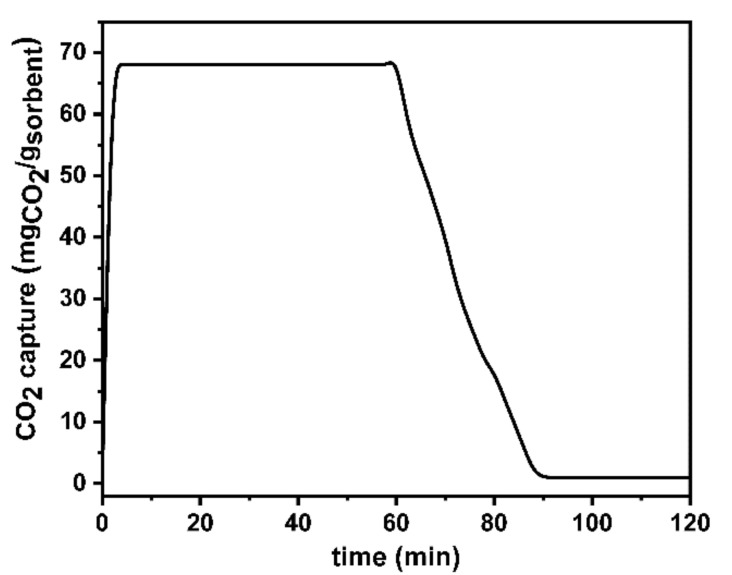
CO_2_ capture by 10 mg of hPPy sample.

The CO_2_ adsorption/desorption capacity of the hPPy membrane, as shown in Figure 7, indicated that the CO_2_ adsorption capacity is ≈68 mg/g. This high affinity of the hPPy is related to the significant number of amino groups. It was reported that the CO_2_ molecules could interact with primary and secondary amines to form carbamate, as represented in Equations (1) and (2) [67,68,69,70,71,72].

CO_2_ + 2RNH_2_ → RNHCO_2_^−^ + RNH_3_^+^ (1)

CO_2_ + 2R_1_R_2_NH → R_1_R_2_NCO_2_^−^ + RR’NH_2_^+^ (2)

On the other hand, the organic heterocyclic molecules that contain N atoms such as pyrrole moiety to interact with CO_2_ through the Lewis acid–Lewis base interactions as well as the hydrogen bonding between the positively charged N atoms of the pyrrole and the negatively charged oxygen atoms of CO_2_ [73,74,75,76,77]. Therefore, the N atoms’ high density within the polymer network increases its adsorbent efficiency toward CO_2_ molecules. Furthermore, the molar ratio of water in the ambient air is typically about 100 times the CO_2_ content. Hence, the development of a selective adsorbent is urgently needed to avoid water co-adsorption [78]. Here, the hydrophobicity characteristic of the hPPy membrane plays a vital role in the CO_2_ adsorption efficiency due to its role to prevent wetting of the membrane pores and thus increase the overall mass transfer coefficient [79]. Figure 7 showed increasing the adsorption of CO_2_ until the pseudoequilibrium was reached after 5 min at 68 mg of CO_2_ for each g of hPPy. The adsorption process was performed for 60 min. Then, the desorption of CO_2_ from the hPPy was studied based on switching the gas flow back into N_2_ at the same temperature. The results showed a linear decrease in the amount of adsorbed CO_2_ until reaching complete desorption within 25 min. This behavior indicated that this system has a completely reversible character. Hence, it showed the possibility of hPPy regeneration without applying heat [77,80]. Furthermore, this regeneration of hPPy could be indicated the existence of weak binding between hPPy and CO_2_. This is one of the advantages during the development of an adsorbent in CO_2_ capture that results in energy-saving regeneration tendency.

## 4. Conclusions

We have developed a new polymer membrane with hollow nanocones morphology in the present work that could be applied in different application fields, including biology, chemistry, and environmental applications. One of the good advantages of this polymer is the easily detachable layer that allows us to use it as a rigid template to fabricate different nanostructures over different materials or as a membrane. Our results demonstrated that we had made a breakthrough in the synthesis of porous conducting polymer membranes. The fabricated polymer showed a large surface area (about 949.5 m^2^/g, the highest yet), a large contact angle of 136.3°, high surface energy, and a pull-off ability as a layer. Furthermore, the fabricated membrane showed high efficiency as a CO_2_ sucker with a gas capacity of about 68 mg/g with a regeneration ability without heat applying. That opens the door for several applications, including biosensors, Li-ion batteries, as hard templates for synthesizing different nanomaterials, drug delivery, membrane, artificial muscle, CO_2_ sucker, etc.

## Figures and Tables

**Table 1:** The surface area of reported polypyrrole and its composites.

Material	Surface Area (m^2^/g)	References
PPy	19.2	55
PPy	10.57	56
PPy/cellulose composite	57	57
Nanocellulose PPy membrane	80	58
PPy hydrogel/Au composites	26.2	59
hPPy	949.5	The present work

## Data Availability

The data presented in this study are available on request from the corresponding author.

## Supplementary Materials

The following supporting information can be downloaded at: https://www.mdpi.com/article/10.3390/polym14010183/s1: Video S1: The movie showed the pull-off of the hollow hPPy nanocones membrane; Video S2−S4: The movies showed the attraction between the hPPy membrane that showed the surface energy.

## References

[1] MacDowell N., Florin N., Buchard A., Hallett J., Galindo A., Jackson G., Adjiman C.S., Williams C.K., Shah N., Fennell P. (2010). An overview of CO_2_ capture technologies. Energy Environ. Sci..

[2] Intergovernmental Panel on Climate Change (IPCC) (2015). Climate Change 2014: Mitigation of Climate Change.

[3] Figueroa J.D., Fout T., Plasynski S., McLlvried H., Srivastava R.D. (2008). Advance in CO_2_ capture technology—The U.S. Department of Energy’s Carbon Sequestration Program. Int. J. Greenh. Gas Control.

[4] Belmabkhout Y., Sayari A. (2009). Effect of pore expansion and amine functionalization of mesoporous silica on CO_2_ adsorption over a wide range of conditions. Adsorption.

[5] Mofijur M., Masjuki H.H., Kalam M.A., Hazrat M.A., Liaquat A.M., Shahabuddin M., Varman M. (2012). Prospects of biodiesel from Jatropha in Malaysia. Renew. Sustain. Energ. Rev..

[6] Yang H., Xu Z., Fan M., Gupta R., Slimane R.B., Bland A.E., Wright I. (2008). Progress in carbon dioxide separation and capture: A review. J. Environ. Sci..

[7] Chen C., Yang S.-T., Ahn W.-S., Ryoo R. (2009). Amine-impregnated silica monolith with a hierarchical pore structure: Enhancement of CO_2_ capture capacity. Chem. Commun..

[8] Choi S., Drese J.H., Jones C.W. (2009). Adsorbent materials for carbon dioxide capture from large anthropogenic point sources. ChemSusChem.

[9] Moura P.A.S., Bezerra D.P., Vilarrasa-Garcia E., Bastos-Neto M., Azevedo D.C.S. (2016). Adsorption equilibria of CO_2_ and CH_4_ in cation-exchanged zeolites 13X. Adsorption.

[10] Ogawa T., Iyoki K., Fukushima T., Kajikawa Y. (2017). Landscape of Research Areas for Zeolites and Metal-Organic Frameworks Using Computational Classification Based on Citation Networks. Materials.

[11] Castrillon M., Moura K.O., Alvez C., Bastos-Neto M., Azevedo D.C.S., Jofnann J., Möller J., Einicke W.-D., Gläser R. (2016). CO_2_ and H_2_S Removal from CH4-Rich Streams by Adsorption on Activated Carbons Modified with K_2_CO_3_, NaOH, or Fe_2_O_3_. Energy Fuels.

[12] Arulkumar M., Thirumalai K., Sathishkumar P., Palvannan T. (2012). Rapid removal of chromium from aqueous solution using novel prawn shell activated carbon. Chem. Eng. J..

[13] Pinto M.L., Pires J. (2012). Porous and hybrid clay based materials for separation of hydrocarbons. Microporous Mesoporous Mater..

[14] Sumida K., Rogow D.L., Mason J.A., McDonald T.M., Bloch E.D., Herm Z.R., Bae T.-H., Long J.R. (2012). Carbon Dioxide Capture in Metal–Organic Frameworks. Chem. Rev..

[15] Donia M., Atia A.A., Daher A.M., Desouky O.A., Elshehy E.A. (2011). Synthesis of Amine/Thiol Magnetic Resin and Study of Its Interaction with Zr(IV) and Hf(IV) Ions in Their Aqueous Solutions. J. Dispers. Sci. Technol..

[16] Bloch W.M., Babarao R., Hill M.R., Doonan C.J., Sumby C.J. (2013). Post-synthetic Structural Processing in a Metal-Organic Framework Material as a Mechanism for Exceptional CO_2_/N_2_ Selectivity. J. Am. Chem. Soc..

[17] Donia A.M., Atia A.A., Daher A.M., Elshehy E.A. (2011). Extraction and Separation of Zirconium(IV) and Hafnium(IV) from Chloride Media Using Magnetic Resin with Phosphoric Acid Functionality. J. Dispers. Sci. Technol..

[18] Fusco C., Casiello M., Catucci L., Comparelli R., Cotugno P., Falcicchio A., Fracassi F., Margiotta V., Moliterni A., Petronella F. (2018). TiO_2_@PEI-Grafted-MWCNTs Hybrids Nanocomposites Catalysts for CO_2_ Photoreduction. Materials.

[19] Vieria R.B., Moura P.A.S., Vilarrasa-Garcia E., Azevedo D.C.S., Pastore H.O. (2018). Polyamine-Grafted Magadiite: High CO_2_ Selectivity at Capture from CO_2_/N_2_ and CO_2_/CH_4_ Mixtures. J. CO2 Util..

[20] Intergovernmental Panel on Climate Change (2006). Carbon Dioxide Capture and Storage.

[21] Hiyoshi N., Yogo K., Yashima T. (2005). Adsorption Characteristics of Carbon Dioxide on Organically Functionalized SBA-15. Microporous Mesoporous Mater..

[22] Mali S.P., Gosavi S.A., Inamdar A.S., Chougale U.M., Fulari V.J. (2015). Synthesis and Characterizations of Chemically and Electrochemically Polymerized Polyaniline Thin Films for Energy Storage. Adv. Sci. Lett..

[23] Zoromba M.S., Al-Hossainy A.F., Abdel-Aziz M.H. (2017). Conductive thin films based on poly (aniline-co-o-anthranilic acid)/magnetite nanocomposite for photovoltaic applications. Synth. Met..

[24] El-Said W.A., Abdelshakour M., Choi J.-H., Choi J.-W. (2020). Application of conducting polymer nanostructures to electrochemical biosensors. Molecules.

[25] El-Said W.A., Yea C.-H., Choi J.-W., Kwon I.-K. (2009). Ultrathin polyaniline film coated on an indium-tin oxide cell-based chip for study of anticancer effect. Thin Solid Films.

[26] Guimard N.K., Gomez N., Schmidt C.E. (2007). Conducting polymers in biomedical engineering. Prog. Polym. Sci..

[27] El-Said W.A., Nasr O., Soliman A.I.A., Elshehy E.A., Khan Z.A., Abdel-Wadood F.K. (2021). Fabrication of polypyrrole/Au nanoflowers modified gold electrode for highly sensitive sensing of paracetamol in pharmaceutical formulation. Appl. Surf. Sci. Adv..

[28] El-Said W.A., Alshitari W., Choi J.-W. (2020). Controlled fabrication of gold nanobipyramids/polypyrrole for shell-isolated nanoparticle-enhanced Raman spectroscopy to detect γ-aminobutyric acid. Spectrochim. Acta—Part A Mol. Biomol. Spectrosc..

[29] Liu X., Wang S. (2014). Three-dimensional nano-biointerface as a new platform for guiding cell fate. Chem. Soc. Rev..

[30] Zhang F., Jiang Y., Liu X., Meng J., Zhang P., Liu H., Yang G., Li G., Jiang L., Wan L.-J. (2016). Hierarchical Nanowire Arrays as Three-Dimensional Fractal Nanobiointerfaces for High Efficient Capture of Cancer Cells. Nano Lett..

[31] Si P., Ding S., Lou X.-W., Kim D.-H. (2011). An electrochemically formed three-dimensional structure of polypyrrole/graphene nanoplatelets for high-performance supercapacitors. RSC Adv..

[32] Chang H.H., Chang C.K., Tsai Y.C., Liao C.S. (2012). Electrochemically synthesized graphene/polypyrrole composites and their use in supercapacitor. Carbon.

[33] Cui Y.M., Wen Z.Y., Liang X., Lu Y., Jin J., Wu M.F., Wu X.W. (2012). A tubular polypyrrole based air electrode with improved O_2_ diffusivity for Li–O_2_ batteries. Energy Environ. Sci..

[34] Ravichandran S., Nagarajan S., Kokil A., Ponrathnam T., Bouldin R.M., Bruno F.F., Samuelson L., Kumar J., Nagarajan R. (2012). Micellar Nanoreactors for Hematin Catalyzed Synthesis of Electrically Conducting Polypyrrole. Langmuir.

[35] Gelmi A., Higgins M.J., Wallace G.G. (2013). Resolving sub-molecular binding and electrical switching mechanisms of single proteins at electroactive conducting polymers. Small.

[36] Song H.S., Kwon O.S., Lee S.H., Park S.J., Kim U.K., Jang J., Park T.H. (2013). Human Taste Receptor-Functionalized Field Effect Transistor as a Human-Like Nanobioelectronic Tongue. Nano Lett..

[37] Xue M.Q., Li F.W., Wang Y., Cai X.J., Pan F., Chen J.T. (2013). Ultralow-limit gas detection in nano-dumbbell polymer sensor viaelectrospinning. Nanoscale.

[38] Ma M.M., Guo L., Anderson D.G., Langer R. (2013). Bio-inspired polymer composite actuator and generator driven by water gradients. Science.

[39] Zheng W., Razal J.M., Spinks G.M., Truong V.T., Whitten P.G., Wallace G.G. (2012). The Role of Unbound Oligomers in the Nucleation and Growth of Electrodeposited Polypyrrole and Method for Preparing High Strength, High Conductivity Films. Langmuir.

[40] Richard Prabakar S.J., Pyo M. (2012). Corrosion protection of aluminum in LiPF6 by poly(3,4-ethylenedioxythiophene) nanosphere-coated multiwalled carbon nanotube. Corros. Sci..

[41] Makris T., Dracopoulos V., Stergiopoulos T., Lianos P. (2011). A quasi solid-state dye-sensitized solar cell made of polypyrrole counter electrodes. Electrochim. Acta.

[42] Bu C., Tai Q., Liu Y., Guo S., Zhao X.J. (2013). A transparent and stable polypyrrole counter electrode for dye-sensitized solar cell. Power Sources.

[43] Zhang J., Liu X.H., Zhang L.X., Cao B.Q., Wu S.H. (2013). Reactive template synthesis of polypyrrole nanotubes for fabricating metal/conducting polymer nanocomposites. Macromol. Rapid Commun..

[44] Lee J.I., Cho S.H., Park S.-M., Kim J.K., Kim J.K., Yu J.-W., Kim Y.C., Russell T.P. (2008). Highly Aligned Ultrahigh Density Arrays of Conducting Polymer Nanorods using Block Copolymer Templates. Nano Lett..

[45] Nam D.-H., Kim M.-J., Lim S.-J., Song I.-S., Kwon H.-S.J. (2013). Single-step synthesis of polypyrrole nanowires by cathodic electropolymerization. Mater. Chem. A.

[46] Massafera M.P., Córdoba de Torresi S.I.J. (2012). Evaluating the performance of polypyrrole nanowires on the electrochemical sensing of ammonia in solution. Electroanal. Chem..

[47] Bai Y., Xu Y., Wang J., Gao M., Wang J. (2014). Interface Effect on the Electropolymerized Polypyrrole Films with Hollow Micro/Nanohorn Arrays. ACS Appl. Mater. Interfaces.

[48] Wang J., Wen Z., Zi Y., Zhou P., Lin J., Guo H., Xu Y., Wang Z.L. (2016). All-Plastic-Materials Based Self-Charging Power System Composed of Triboelectric Nanogenerators and Supercapacitors. Adv. Funct. Mater..

[49] Ma S., Hu S., Wang Q., Liu Y., Zhao G., Zhang Q., Mao C., Zhao B. (2017). Evaluation of sialic acid based on electrochemical cytosensor with 3D micro/nanostructured sensing interface. Anal. Methods.

[50] Song J., Liu H., Wan M., Zhu Y., Jiang L.J. (2013). Bio-inspired isotropic and anisotropic wettability on a Janus free-standing polypyrrole film fabricated by interfacial electro-polymerization. Mater. Chem. A.

[51] Santos L., Martin P., Ghilane J., Lacaze P.C., Lacroix J.-C. (2013). Micro/Nano-Structured Polypyrrole Surfaces on Oxidizable Metals as Smart Electroswitchable Coatings. ACS Appl. Mater. Interfaces.

[52] Tang Y.H., Wu N., Luo S.L., Liu C.B., Wang K., Chen L.Y. (2012). One-Step Electrodeposition to Layer-by-Layer Graphene–Conducting-Polymer Hybrid Films. Macromol. Rapid Commun..

[53] Wang J.P., Xu Y.L., Wang J., Du X.F., Xiao F., Li J.B. (2010). High charge/discharge rate polypyrrole films prepared by pulse current polymerization. Synth. Met..

[54] Choi J.H., El-Said W.A., Choi J.W. (2020). Highly sensitive surface-enhanced Raman spectroscopy (SERS) platform using core/double shell (Ag/polymer/Ag) nanohorn for proteolytic biosensor. Appl. Surf. Sci..

[55] Sharma M., Waterhouse G.I.N., Loader S.W.C., Garg S., Svirskis D. (2013). High surface area polypyrrole scaffolds for tunable drug delivery. Int. J. Pharm..

[56] Olatunji M.A., Khandaker M.U., Amin Y.M., Ekramul Mahmud H.N.M. Development and Characterization of Polypyrrole-Based Nanocomposite Adsorbent and Its Applications in Removal of Radioactive Materials. Proceedings of the International Conference for Innovation in Biomedical Engineering and Life Sciences (ICIBEL 2015).

[57] Mihranyan A., Nyholm L., Bennett A.E.G., Strømme M.J. (2008). A Novel High Specific Surface Area Conducting Paper Material Composed of Polypyrrole and Cladophora Cellulose. Phys. Chem. B.

[58] Ferraz N., Carlsson D.O., Hong J., Larsson R., Fellström B., Nyholm L., Strømme M., Mihranyan A.J.R. (2012). Haemocompatibility and ion exchange capability of nanocellulose polypyrrole membranes intended for blood purification. J. R. Soc. Interface.

[59] Rong Q., Han H., Feng F., Ma Z. (2015). Network nanostructured polypyrrole hydrogel/Au composites as enhanced electrochemical biosensing platform. Sci. Rep..

[60] Sing K.S.W., Everett D.H., Haul R.A.W., Moscou L., Pierotti R.A., Rouquerol J., Siemieniewska T. (1985). Reporting physisorption data for gas/solid systems with special reference to the determination of surface area and porosity (Recommendations 1984). Pure Appl. Chem..

[61] Broekhoff J.C.P. (1979). Mesopore determination from nitrogen sorption isotherms: Fundamentals, scope, limitations. Stud. Surf. Sci. Catal..

[62] Shields J.E., Lowell S., Thomas M.A., Thommes M. (2004). Characterization of Porous Solids and Powders: Surface Area, Pore Size and Density.

[63] Adhikari S., Sarkar D., Madras G. (2017). Hierarchical Design of CuS Architectures for Visible Light Photocatalysis of 4-Chlorophenol. ACS Omega.

[64] Partch R., Gangolli S.G., Matijević E., Cal W., Arajs S. (1991). Conducting polymer composites: I. Surface-induced polymerization of pyrrole on iron(III) and cerium(IV) oxide particles. J. Colloid Interface Sci..

[65] Emran K.M., Ali S.M., Al-Oufi A.L.L. (2017). Synthesis and Characterization of Nano-Conducting Copolymer Composites: Efficient Sorbents for Organic Pollutants. Molecules.

[66] Sampathkumar L., Selvin P.C., Selvasekarapandian S., Perumal P., Chitra R., Muthukrishnan M. (2019). Synthesis and characterization of biopolymer electrolyte based on tamarind seed polysaccharide, lithium perchlorate and ethylene carbonate for electrochemical applications. Ionics.

[67] McCann N., Phan D., Fernandes D., Maeder M. (2011). A Systematic Investigation of Carbamate Stability Constants by ^1^H NMR. Int. J. Greenh. Gas Control..

[68] Sanz R., Calleja G., Arencibia A., Sanz-Pèrez E.S. (2010). CO_2_ Adsorption on Branched Polyethyleneimine-Impregnated Mesoporous silica SBA-15. Appl. Surf. Sci..

[69] Aresta M., Quaranta E. (1992). Role of the Macrocyclic Polyether in the Synthesis of N-Alkylcarbamate Esters from Primary Amines, CO_2_ and Alkyl Halides in the Presence of Crown-Ethers. Tetrahedron.

[70] Wang X.X., Schwartz V., Clark J.C., Ma M.L., Overbury S.H., Xu X.C., Song C.S. (2009). Infrared Study of CO_2_ Sorption over “Molecular Basket” Sorbent Consisting of Polyethylenimine-Modified Mesoporous Molecular Sieve. J. Phys. Chem..

[71] White C.M., Strazisar B.R., Granite E.J., Hoffman J.S. (2003). Separation and capture of CO_2_ from large stationary sources and sequestration in geological formations--coalbeds and deep saline aquifers. J. Air Waste Manag. Assoc..

[72] Chang A.C.C., Chuang S.S.C., Gray M., Soong Y. (2003). In-Situ Infrared Study of CO_2_ Adsorption on SBA-15 Grafted with y-(Aminopropyl)triethoxysilane. Energy Fuels.

[73] Vogiatzis K., Mavrandonakis A., Klopper W., Froudakis G.E. (2009). Ab initio study of the interactions between CO_2_ and N-containing organic heterocycles. ChemPhysChem.

[74] Du N., Park H.B., Robertson G.P., Dal-Cin M.M., Visser T., Scoles L., Guiver M.D. (2011). Polymer nanosieve membranes for CO_2_-capture applications. Nat. Mater..

[75] Nabavi S.A., Vladisavljević G.T., Zhu Y., Manović V. (2017). Synthesis of size-tunable CO_2_-philic imprinted polymeric particles (MIPs) for low-pressure CO_2_ capture using oilin-oil suspension polymerization. Environ. Sci. Technol..

[76] Azofra L.M., Altarsha M., Ruiz-López M.F., Ingrosso F. (2013). A theoretical investigation of the CO_2_-philicity of amides and carbamides. Theor. Chem. Acc..

[77] Fayemiwo K.A., Vladisavljević G.T., Nabavi S.A., Benyahia B., Hanak D.P., Loponov K.N., Manović V. (2018). Nitrogen-rich hyper-crosslinked polymers for low-pressure CO_2_ capture. Chem. Eng. J..

[78] Lackner K.S. (2009). Capture of carbon dioxide from ambient air. Eur. Phys. J.—Spec. Top..

[79] Goyal N., Suman S., Gupta S.K. (2015). Mathematical modeling of CO_2_ separation from gaseous-mixture using a Hollow-Fiber Membrane Module: Physical mechanism and influence of partial-wetting. J. Membr. Sci..

[80] Mane S., Gao Z.-Y., Li Y.-X., Xue D.-M., Liu X.-Q., Sun L.-B. (2017). Fabrication of microporous polymers for selective CO_2_ capture: The significant role of crosslinking and crosslinker length. J. Mater. Chem. A.

